# A Novel Multidrug-Resistant Cell Line from an Italian Intrahepatic Cholangiocarcinoma Patient

**DOI:** 10.3390/cancers13092051

**Published:** 2021-04-23

**Authors:** Caterina Peraldo-Neia, Annamaria Massa, Francesca Vita, Marco Basiricò, Chiara Raggi, Paola Bernabei, Paola Ostano, Laura Casorzo, Mara Panero, Francesco Leone, Giuliana Cavalloni, Massimo Aglietta

**Affiliations:** 1 Laboratory of Cancer Genomics, Fondazione Edo ed Elvo Tempia, 13900 Biella, Italy; caterina.peraldoneia@fondazionetempia.org (C.P.-N.); paola.ostano@fondazionetempia.org (P.O.); 2 Division of Medical Oncology, Candiolo Cancer Institute, FPO-IRCCS, 10060 Candiolo (Torino), Italy; annamaria.massa@ircc.it (A.M.); marco.basirico@ircc.it (M.B.); massimo.aglietta@ircc.it (M.A.); 3 Department of Oncology, University of Torino, 10126 Torino, Italy; francesca.vita@ircc.it; 4 Department of Experimental and Clinical Medicine, University of Firenze, 50134 Firenze, Italy; chiara.raggi@unifi.it; 5 Flow Cytometry Center, Candiolo Cancer Institute, FPO-IRCCS, 10060 Candiolo (Torino), Italy; paola.bernabei@ircc.it; 6 Unit of Pathology, Candiolo Cancer Institute, FPO-IRCCS, 10060 Candiolo (Torino), Italy; laura.casorzo@ircc.it (L.C.); mara.panero@ircc.it (M.P.); 7 Department of Oncology, Nuovo Ospedale degli Infermi, Azienda Sanitaria Locale Biella, 13875 Ponderano (Biella), Italy; francesco.leone@aslbi.piemonte.it

**Keywords:** intrahepatic cholangiocarcinoma, new cell line, patient-derived in vitro model

## Abstract

**Simple Summary:**

Intrahepatic cholangiocarcinoma (ICC) has limited prognosis and therapies. The first-line gemcitabine-based therapy provided poor benefits in terms of survival due to the development of resistance. Gemcitabine-resistance mechanisms were studied on pancreatic cancer models or cell lines derived from ICC patients of Eastern countries. Since ICC has different etiology and genetic/molecular characteristics depending on the ethnicity, appropriate preclinical models that recapitulate their biology are required. Thus, we aimed to establish and characterize an Italian ICC cell line, named 82.3. Cells were isolated from a patient-derived xenograft. After one year, immunophenotypical, biological, genetic, molecular features, and in vivo tumorigenicity in NOD/SCID mice were investigated. Furthermore, 82.3 cells displayed resistance to gemcitabine, 5-fluorouracil, carboplatin, and oxaliplatin. This model could be exploited either to investigate drug resistance mechanisms or to test alternative drugs through the identification of suitable targets to overcome drug resistance.

**Abstract:**

Chemotherapy resistance is a relevant clinical issue in tumor treatment, in particular in biliary tract carcinoma (BTC), for which there are no effective therapies, neither in the first nor in the second line. The development of chemoresistant cell lines as experimental models to investigate the mechanisms of resistance and identify alternative druggable pathways is mandatory. In BTC, in which genetics and biological behavior depend on the etiology, ethnicity, and anatomical site of origin, the creation of models that better recapitulate these characteristics is even more crucial. Here we have established and characterized an intrahepatic cholangiocarcinoma (iCCA) cell line derived from an Italian patient, called 82.3. Cells were isolated from a patient-derived xenograft (PDX) and, after establishment, immunophenotypic, biological, genetic, molecular characteristics, and tumorigenicity in vivo in NOD/SCID mice were investigated. 82.3 cells exhibited epithelial morphology and cell markers (EPCAM, CK7, and CK19); they also expressed different cancer stem markers (CD44, CD133, CD49b, CD24, Stro1, PAX6, FOXA2, OCT3/4), α–fetoprotein and under anchorage-independent and serum-free conditions were capable of originating cholangiospheres. The population doubling time was approximately 53 h. In vitro, they demonstrated a poor ability to migrate; in vivo, 82.3 cells retained their tumorigenicity, with a long latency period (16 weeks). Genetic identity using DNA fingerprinting analysis revealed 16 different loci, and the cell line was characterized by a complex hyperdiploid karyotype. Furthermore, 82.3 cells showed cross-resistance to gemcitabine, 5-fluorouracil, carboplatin, and oxaliplatin; in fact, their genetic profile showed that 60% of genes (*n* = 168), specific for drug resistance and related to the epithelial-mesenchymal transition, were deregulated in 82.3 cells compared to a control iCCA cell line sensitive to chemotherapeutics. RNA sequencing analysis revealed the enrichment for genes associated with epithelial to mesenchymal transition (EMT), vasculature development, and extracellular matrix (ECM) remodeling, underlining an aggressive phenotype. In conclusion, we have created a new iCCA cell line of Caucasian origin: this could be exploited as a preclinical model to study drug resistance mechanisms and to identify alternative therapies to improve the prognosis of this tumor type.

## 1. Introduction

Intrahepatic cholangiocarcinoma (iCCA) is a rare and aggressive liver neoplasm originating from the cholangiocytes of the intrahepatic biliary duct branches tract [1]. Its incidence accounts for 10–20% of all biliary tract carcinoma (BTC), and its prognosis is dismal; surgery is potentially curative only at the early stage, while survival for the advanced iCCA patients reaches 5 years in less than 10% [2]. Chemotherapy approaches, based on gemcitabine in association with platinum derivatives [3], as well as target or radiotherapy, have provided limited benefits in terms of survival. Moreover, although BTC is more susceptible to gemcitabine compared to other chemotherapeutic agents, patients could present intrinsic or develop acquired resistance, which impairs its efficacy [4].

Several mechanisms responsible for intrinsic and acquired gemcitabine resistance have been identified, mainly in pancreatic preclinical models; they include downregulation of gemcitabine transporter, alterations of proteins involved in gemcitabine catabolism, activation of alternative pathways in DNA repair, resistance to apoptosis, and promotion of epithelial to mesenchymal transition (EMT). The standard regimen for gemcitabine-resistant patients is not well established; however, a portion of patients progressing during gemcitabine treatment may receive fluoropyrimidine-based chemotherapy as second-line [5,6,7,8]. Although not conclusive, clinical data indicated that patients treated with gemcitabine-cisplatin regimen as first-line, followed by fluoropyrimidine-based chemotherapy as second-line showed an overall response rate of 3% and a median progression-free survival of 1.9 months (95% confidence interval (CI), 1.6–2.2) [8]. These data may suggest a cross-resistance to both drugs, as already demonstrated in preclinical models of pancreatic cancer and BTC [9,10]. Thus, it is mandatory to find alternative therapeutic options for these multidrug-resistant patients.

To achieve this goal, appropriate preclinical models are required. To date, cell lines originating from BTC primary tumors are useful to explore biology and cholangiocarcinogenesis, to test drug sensitivity, to develop molecular therapeutic targets, and to study drug resistance mechanisms.

Taking into account the different etiology of BTCs and their genetic aberrations associated with both etiology and ethnicity [11], it is important to use appropriate preclinical models that reflect these characteristics. To date, most of the established and characterized cell lines are of Eastern origin.

Here, we established and biologically and molecularly characterized a new iCCA cell line, named 82.3, derived from an Italian patient. This is a multidrug-resistant in vitro model that could be exploited to investigate drug resistance mechanisms, to identify druggable specific pathways, to find effective alternative chemotherapeutic agents, and to overcome or to revert resistance.

## 2. Results

### 2.1. Generation and Immunophenotyping of ICC Cell Line

**Figure 1:**
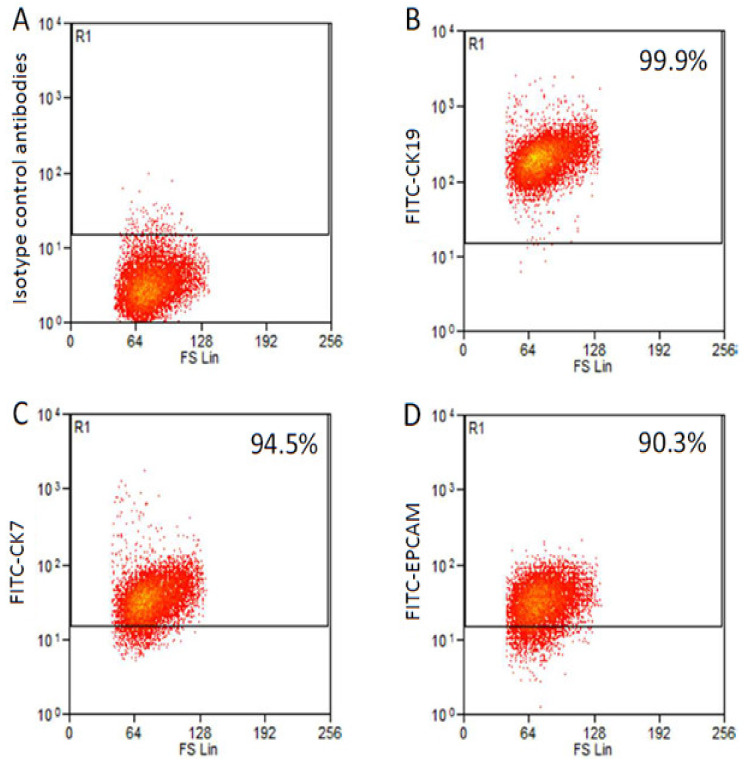
Immunophenotypic analysis of 82.3 cells. Isotype control: cells incubated with antibody isotype as primary antibody (**A**). Epithelial cell markers CK19 (99.9%) (**B**), CK7 (94.5%) (**C**), and EPCAM (90.3%) (**D**).

**Figure 2:**
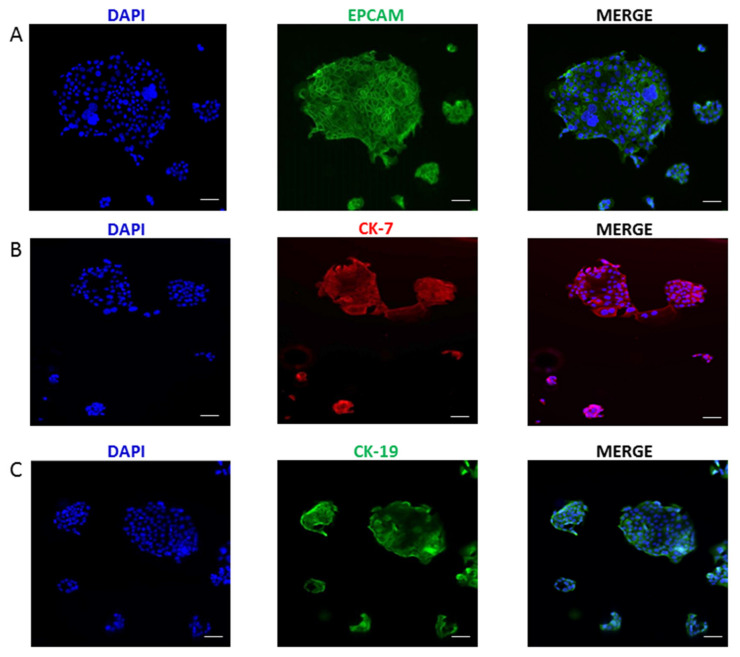
Immunofluorescence staining of CCA markers in 82.3 cells. EPCAM (Alexa Fluor 488) (**A**), CK-7 (Alexa Fluor 647) (**B**), and CK-19 (Alexa Fluor 488) (**C**). Nuclei were counterstained in blue (DAPI) (magnification 40×).

Cells were isolated after the dissociation of the tumor mass derived from a 4th generation PDX. After two months in the optimal cell culture conditions, cells were detached for the first time. After about 1 year, a stable cell line was obtained and named 82.3. Immunophenotypical analysis revealed that 82.3 cells expressed biliary epithelial cell markers, such as EPCAM (90.3%), CK7 (94.5%), and CK19 (99.9%), as shown in Figure 1 and Figure 2.

The same levels of marker expression were observed in 82.3 cells at early passages (p10) (Figure S1). Moreover, we observed that 82.3 cells expressed α-fetoprotein (AFP) (Figure S2), a marker of hepatocellular carcinoma, sometimes detected in BTC [12].

### 2.2. Morphological, Phenotypic, and Biological Characterization of 82.3 Cells

**Figure 3:**
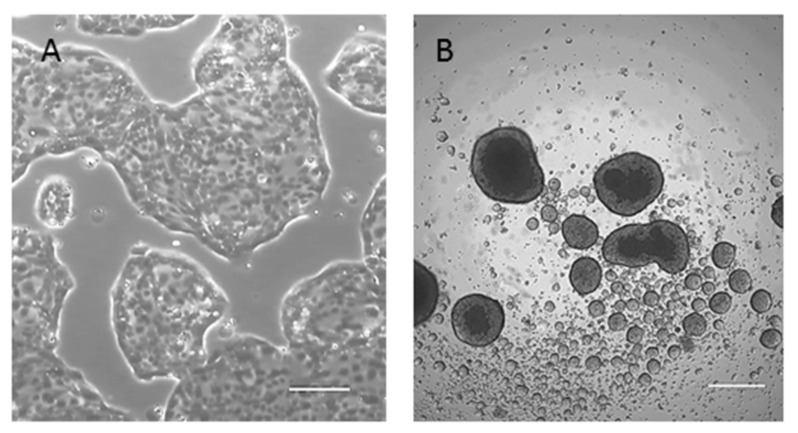
Morphology of 82.3 cells. (**A**) Cells were able to grow in monolayer and exhibited epithelial morphology (4× magnification); (**B**) representative images of cholangiospheres of 82.3. Cells were seeded in ultra-low attachment plate and stem cell-serum-free medium. Sphere formation was monitored on days 7, 10, and 14 after seeding (4× magnification).

82.3 cells, in optimal culture conditions, appeared as a homogeneous culture with epithelial morphological features (Figure 3A). They were also able to grow in an anchorage-independent manner and to originate well-structured cholangiospheres (Figure 3B) in low-attachment conditions and in stem cell-serum-free medium, suggesting stemness features.

**Figure 4:**
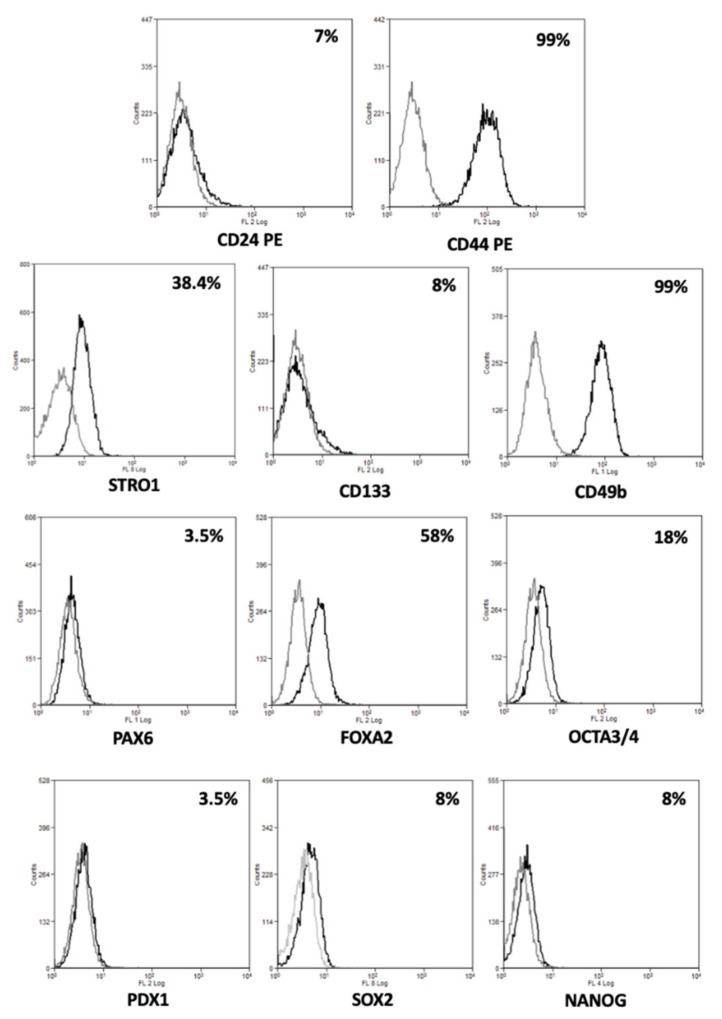
Expression of stem markers in 82.3 cells. Representative histograms of percentage of 82.3 positive cells for surface (CD24, CD44, CD133, CD49b, and Stro1), and intracellular stem markers (PAX6, FOXA2, OCTA3/4, PDX1, SOX2, and NANOG).

To confirm their stem cell status, we investigated the expression of several stemness and pluripotency markers. As shown in Figure 4, 82.3 cells expressed CD24 (7%), CD44 (99%), Stro1 (38.4%), CD133 (8%), and CD49b (99%) surface stem markers and, PAX6 (3.5%), FOXA2 (58%), OCTA3/4 (18%), PDX1 (3.5%), SOX2 (8%), and NANOG (8%) intracellular stem markers.

**Figure 5:**
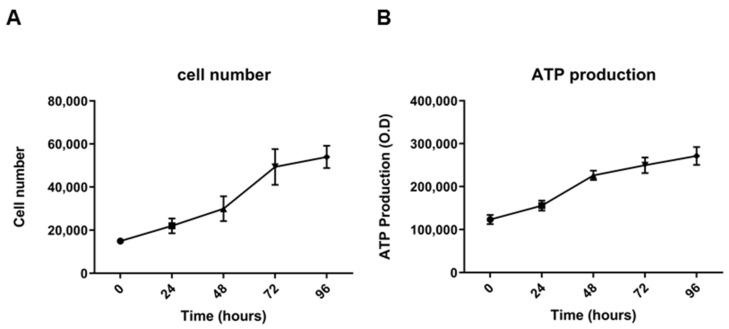
Growth curves of 82.3 cells. Viable cells were counted at 24, 48, 72, and 96 h after seeding (**A**). ATP production at 24, 48, 72, and 96 h of culture after seeding, obtained by CellTiter GLO^®^ assay of 82.3 cells (**B**).

The population doubling time was about 53 h when cultured in a monolayer and in an appropriate culture medium for 96 h (Figure 5A). The same trend was obtained by quantifying ATP production, a key indicator of metabolically active cells (Figure 5B). Cell growth was also assessed at early passage (p10). In this case, the population doubling time was about 48 h (Figure S3).

**Figure 6:**
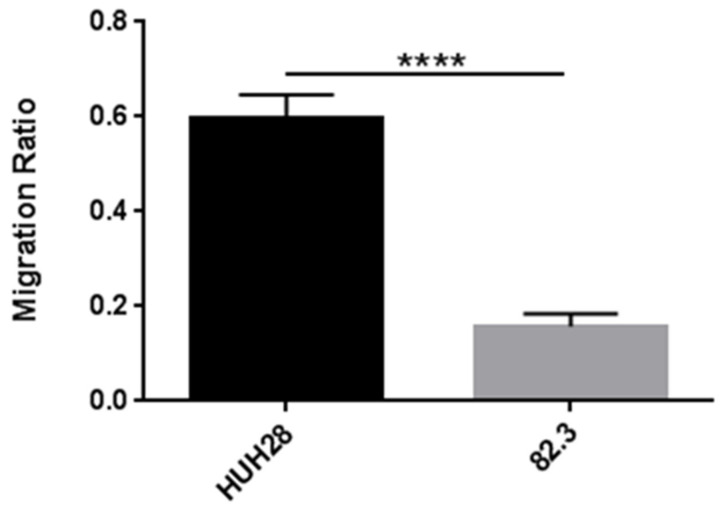
Transwell migration assay on 82.3 and HUH28 cells. Cells were seeded on the surface of the migration transwell chamber and separated by a porous membrane. After 48 h of incubation, the membranes were fixed with methanol and stained with crystal violet. The area of the cells that invaded the membrane was calculated using the ImageJ 2 software; five different fields were evaluated. Migration is expressed as the ratio of the mean ± SEM of the area of migrated cells to the area of the same number of cells plated (control). **** *p* < 0.00001.

The transwell migration assay revealed that, even after 48 h, 82.3 cells had a poor migration potential when compared to the control cell line of iCCA, the HUH28 (Figure 6).

**Figure 7:**
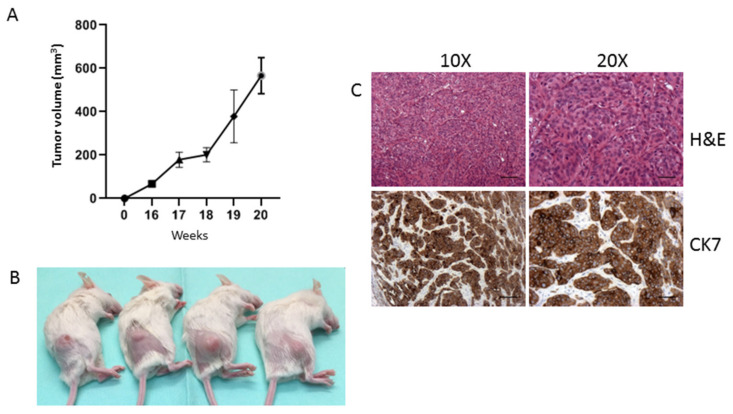
Tumor formation in NOD/SCID mice inoculated with 82.3 cells. In vivo tumor growth curve of 82.3 cells (**A**). Representative images of tumors derived from the injection of 5 × 10^6^ of 82.3 cells (**B**). Representative histochemistry and immunohistochemistry images for the CK7 expression of explanted tumors acquired at 10× and 20× magnification. Scale bar: 100 µm (**C**).

To verify whether 82.3 cells still retained tumorigenic capacity in vivo, they were subcutaneously inoculated into NOD/SCID mice; tumor growth was monitored weekly. A total of six out of six mice developed tumors, but, as shown in Figure 7, the latency period is approximately 16 weeks. Between the 17th and 18th week, all mice reached a homogenous tumor volume of about 200 mm^3^; however, at the 19th week, an average volume of 350 mm^3^ was reached but with high variability, ranging from 164 to 693 mm^3^. At the 20th week, the variability decreased, and the mean volume ranged from 425 to 773 mm^3^. Figure 7C shows the epithelial nature of the explanted tumors, as demonstrated by the presence of CK7.

### 2.3. Genetic and Molecular Characterization of Cells 82.3

To analyze the genetic identity of 82.3 cells, DNA fingerprinting was performed. Several highly polymorphic DNA markers covering 16 STR loci validated at different chromosomal locations were included in this analysis (Table 1).

**Figure 8:**
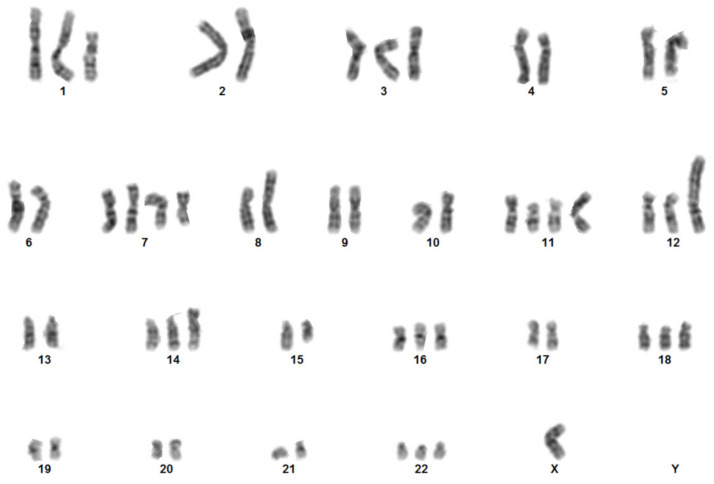
Chromosome analysis: Representative karyotype obtained by G-banding of 82.3 cells. Aberrations were described according to the International System for Human Cytogenetic Nomenclature, 2016.

To further characterize the 82.3 cells, the karyotype analysis was carried out using G−banding. The cytogenetic analysis on 22 metaphases showed an extremely complex karyotype with hyperdiploidy and hypertriploidy. Three different clones have been identified with the following chromosomal rearrangements: Hyperdiploid clone: 50~57,X,−Y,+del(1)(p21p36),+3,−6,+7,+i(7)(p10),add(8)(p22),t(9;15)(p23;q21),der(9)t(9;15)(p23;q21),+del(11)(p12p15),+der(11;13)(q10;q10),+del(12)(p12),+der(12)t(2;12)(q11;p12),+add(14)(p11),−15,+16,+18,+20,+22[cp16]; Hypotriploid clone: 58~62,X,−X,−Y,del(1)(p21p36),+3,−4,−5,−6,+i(7)(p10),−8,add(8)(p22),t(9;15)(p23;q21)x2,del(11)(p12p15)x2,+der(11;13)(q10;q10),del(12)(p12)x2,+der(12)t(2;12)(q11;p12),−14,−21[cp3]; diploid-like clone: 45~49,X,−Y,del(1)(p21p36),+3,−6,+7,+i(7)(p10),−8,t(9;15)(p23;q21),del(11)(p12),der(11;13)(q10;q10),del(12)(p12),der(12)t (2;12)(q11;p12),+20,+22[cp3]. Figure 8 shows a representative karyotype; in contrast with the result obtained by DNA fingerprinting, in which the Y chromosome is detected, in the karyotype the Y is not identifiable, probably due to the numerous translocations.

### 2.4. Multidrug Resistance of 82.3

**Figure 9:**
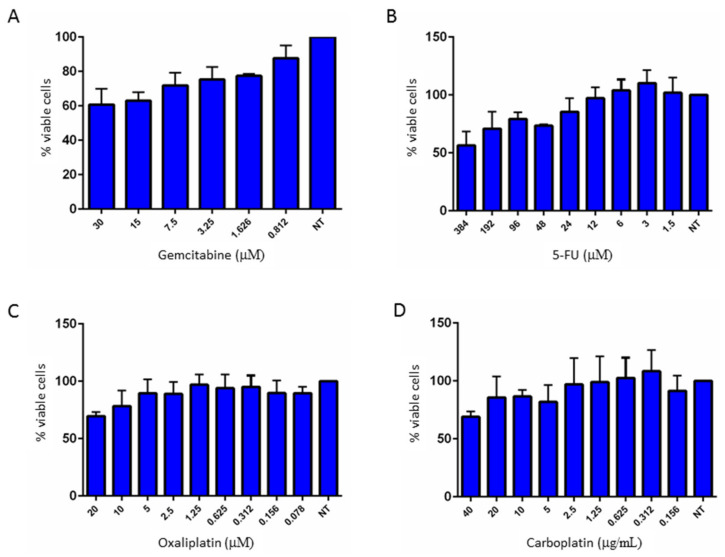
Dose-effect graphs of gemcitabine (**A**), 5-FU (**B**), oxaliplatin (**C**), and carboplatin (**D**) in 82.3 cells. The cells were treated with the indicated scalar doses of the drugs, and cell growth was evaluated after 72 h of incubation using Cell Titer-Glo^®^ (Promega, Milan, Italy). NT: not treated.

The antitumor activity of gemcitabine and 5-FU on cell proliferation was evaluated in 82.3 cells, using escalating doses of these drugs. As shown in Figure 9A,B, 82.3 cells were resistant to both drugs, even at high doses.

To check whether 82.3 cells were also resistant to other chemotherapy drugs, the antiproliferative activity of oxaliplatin and carboplatin was evaluated. As shown in Figure 9C,D, 82.3 cells were poorly responsive to both drugs, often associated with gemcitabine in the first line of treatment in BTC.

**Figure 10:**
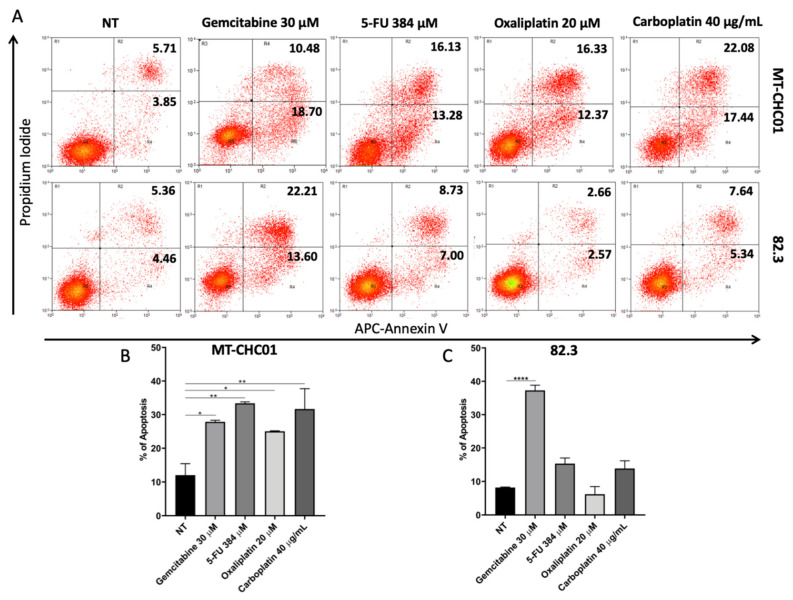
Evaluation of apoptosis after drug treatment in 82.3 and MT-CHC01 cells by Annexin V/Propidium Iodide assay. Cells were treated with the indicated doses of the drugs, and apoptosis was evaluated after 72 h. Flow charts report the percentage of early and late apoptosis (**A**). Graphs represent the percentage of all apoptotic events in MT-CHC01 (**B**) and 82.3 (**C**) cells. NT: not treated. * *p* < 0.01, ** *p* < 0.001 and **** *p* < 0.00001.

Apoptosis was analyzed upon treatment with the above-mentioned drugs at higher concentration after 72 h by Annexin V/PI staining (Bender MedSystems, Wien, Austria) the MT-CHC01 drug-sensitive cell line was used as control. Figure 10 showed that 5-FU, oxaliplatin, and carboplatin did not significantly affect the apoptosis rate of 82.3 cells. Surprisingly, gemcitabine induced about 35% of apoptosis, G1 cell fraction is increased, and S and G2 fraction decreased, this data could also be explained by the high heterogeneity of cell population observed by karyotyping.

### 2.5. Modulation of Genes Related to Epithelial to Mesenchymal Transition (EMT) and Drug Resistance (DR)

82.3 cells were found to be resistant ab initio to different chemotherapeutic agents, suggesting that genes and mechanisms involved in drug resistance and aggressiveness were deregulated. To identify which genes are involved in the resistance process and in the development of a more aggressive phenotype, the mRNA expression of 168 genes known to be involved in drug resistance (DR) and in epithelial-mesenchymal transition (EMT) was quantified. In the absence of the 82.3 parental sensitive clones, the MT-CHC01 cell line was chosen as a control for drug responsiveness. From the DR panel (Table 2), 49 genes were modulated; of them, 23 were up-regulated and 26 were down-regulated. Among the most up-regulated, we found *AHR/ARNT, APC, BRCA2, CDKN1A CYP2E1, HIF1A, NAT2, PPARG, TPMT, UGCG, TOP2B*. The overexpression of some of these genes agrees with that described in other types of tumor [13,14,15,16,17]. As concern the EMT panel (Table 3), 50 of the 84 investigated genes were altered; in particular, 34 are up-regulated, and 16 are down-regulated. Among the most overexpressed, *CAMK2N1, DSC2, FN1, MMP9, RAC1, TGFB2, TMEFF1, VCAN*, and *VIM,* some of them have already been described to be up-regulated in tumor models with more aggressive phenotypes. Of note, there was a significant increase in *VIM* mRNA, a typical mesenchymal marker, accompanied by a decrease in the expression of *KRT19* and *KRT7* epithelial markers [11,13,14,15,16,17].

### 2.6. Transcriptomic Profile of 82.3 Cells

**Figure 11:**
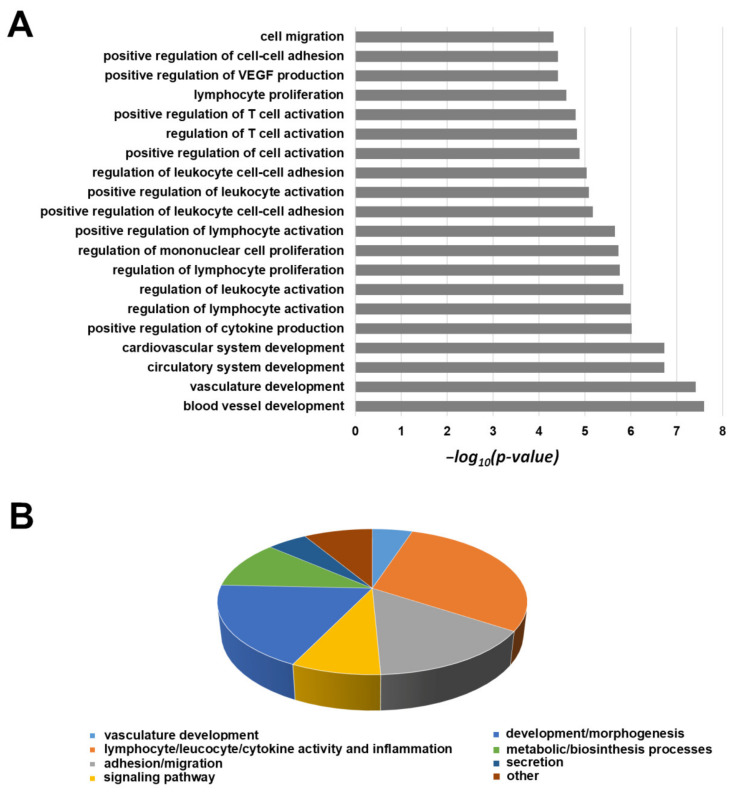
The first 20 GO biological processes (level 5) overrepresented within the list of up-regulated transcripts (**A**). Distribution of all significantly enriched biological processes (*p*-value < 0.01) (**B**).

In order to better clarify the molecular features and the altered pathways in 82.3 cells, we compared the mRNA profile with that of MT-CHC01 cells. The transcriptomic analysis revealed that 1406 genes are deregulated in 82.3 compared to MT-CHC01 cells (Table S1). Since the high number of differentially expressed genes, we used the first 500 up- and 500 down-regulated for the subsequent analyses. As the first step, gene ontology was carried out using the DAVID tool v6.8 (https://david.ncifcrf.gov/). Considering only up-regulated genes, we found enrichment for genes involved in vasculature development, lymphocytes, leukocytes, T cell proliferation and activation, cell migration, and adhesion processes. Figure 11A represented the first 20 biological processes (BP) overrepresented, and Figure 11B showed a sub-classification of all the biological processes with a *p*-value < 0.01.

**Figure 12:**
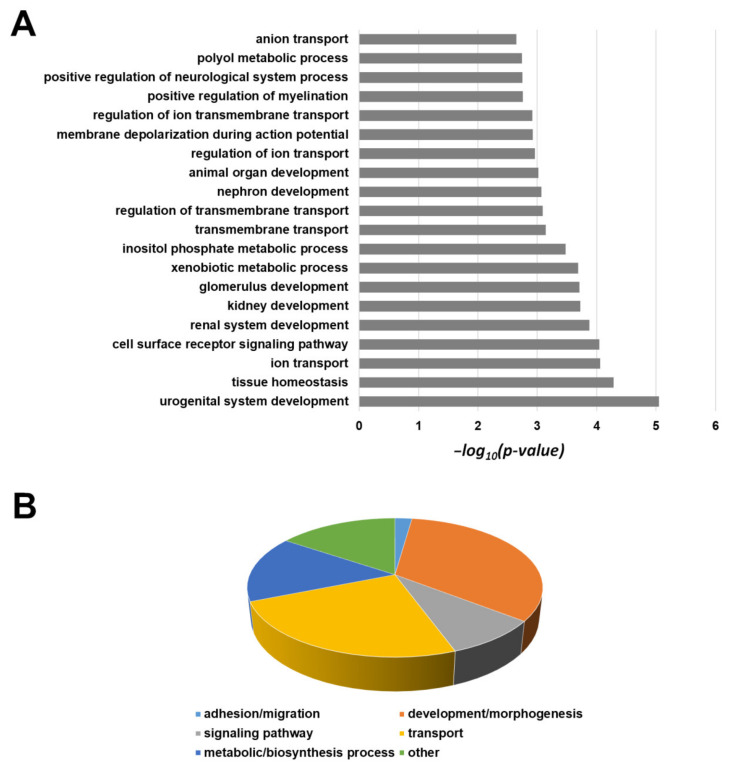
The first 20 GO biological processes (level 5) overrepresented within the list of down-regulated transcripts (**A**). Distribution of all significantly enriched biological processes (*p*-value < 0.01) (**B**).

The same gene panel was used for pathway maps and process networks analyses by Metacore software, version 21.1(Tables S2A and S2B, respectively); the immune response, inflammation, EMT pathways are overrepresented, while the most significant process networks are related to connective tissue degradation and ECM remodeling. The same procedure was conducted for down-regulated genes, revealing an enrichment of ion transport, biosynthesis, and metabolic processes, in particular, xenobiotic, different tissues development, signaling pathway regulation, as shown in Figure 12A (first 20 BP) and Figure 12B (overrepresented subclasses with *p* < 0.01).

The enriched pathway maps and process networks for down-regulated genes are summarized in Table S3A,B; of interest, we found cytoskeleton remodeling, regulation of xenobiotic and bile acid metabolism, proteolysis, cell adhesion, and ESR1 signaling.

## 3. Discussion

Biliary tract carcinomas are heterogeneous tumors with different incidence; in Italy, the incidence rate is gradually increasing, in particular for iCCAs. The heterogeneity of these tumors also extends to the genetic and molecular profiles, which, in turn, are associated with ethnicity and etiology. Conventional therapeutic approaches, based on gemcitabine plus platinum or 5-FU, alone or in association with target therapies, provide poor results in terms of progression-free survival and overall survival [3,4].

The use of preclinical models that reflect the biological characteristics of a particular ethnicity and histotype of CCA is essential for (i) the study of molecular pathogenesis, (ii) the identification of potential molecular targets, (iii) the identification of mechanisms of resistance to standard chemotherapy, and (iv) the alternative to overcome or revert such resistance.

To date, preclinical models are limited, and the in vitro models are mainly represented by cell lines derived from Eastern patients. The MT-CHC01 cell line, stabilized in our laboratory from an Italian patient 4 years ago, was the first European model of iCCA [11].

Here, we have stabilized and characterized a new iCCA cell line, the 82.3, derived from a PDX of an Italian patient. These cells autonomously immortalized after 60 passages, which corresponded at about one year from the dissociation.

They grew in adhesion if cultured in the appropriate medium and presented an epithelial morphology; 82.3 cells expressed high levels of EPCAM, CK7, and CK19, confirming their biliary origin, as demonstrated by flow cytometry and immunofluorescence. Furthermore, we identified that 82.3 cells expressed AFP, a key biomarker of hepatocellular carcinoma [18]. In some cases, AFP is also expressed in BTC; recently, some studies correlate its expression with a worse prognosis [12,19]. Notably, Ishii et al. proposed AFP as a new marker of cancer stem cell progenitor in BTC [20]. Regarding the biological features, 82.3 cells have a duplication time of approximately 53 h and poor migration potential, reflecting the reduced metastatic potential of iCCAs. Under anchorage-independent conditions and in a specific stem cell medium, in the absence of serum, the 82.3 cells form well-defined spheroid structures, suggesting the presence of a stem cell compartment. The analysis of the expression of both surface and intracytoplasmic stem markers, characteristic of biliary cancer stem cells [21], supports the presence of putative cancer stem cells. In particular, there is a high expression of CD44, CD49b (99% of cells are positive), and 18, 38.4, and 58% of cells express OCTA3/4, Stro 1, and FOXA2, respectively.

82.3 cells also maintained the ability to induce tumors in immunodeficient mouse models, with an efficiency of 100%; however, the long latency period does not make 82.3 cells the most suitable model for in vivo experiments. Analysis of DNA fingerprinting on 16 different loci showed that STRs were found in the following alleles: Amelogenin: X; CSF1PO: 11; D13S317: 9.10; D16S539: 11; D18S51: 13.16; D21S11: 30; D3S1358: 14.15; D5S818: 12; D7S820: 8.9; D8S1179: 13.14; FGA: 23; PentaD: 9; PentaE: 10.16; THO1: 6.9; TPOX: 9; vWA: 18.

From the cytogenetic point of view, 82.3 cells exhibit a complex karyotype; the cell population is represented by three different clones with different chromosomal structures: a hyperdiploid, a hypotriploid, and a diploid-like clone, each characterized by structural and numerical anomalies affecting different chromosomes. Moreover, the presence of three different clones well represented the complex intratumoral heterogeneity of iCCA, suggesting that drug treatments could favor the selection of more resistant clones. In fact, the main issue of chemotherapy is the development of chemoresistance and, in particular, cross-resistance to multiple drugs, in particular to gemcitabine and 5-FU in CCA [22]. The 82.3 cells were resistant to gemcitabine, 5-FU, carboplatin, and oxaliplatin, as demonstrated by cell viability assay. The result was confirmed by the evaluation of apoptosis induction with the exception of gemcitabine treatment, by which about 35% of apoptotic cells were observed, underlining the complex and heterogeneous population of this cell line.

Several pathways and genes involved in chemoresistance, validated in in vitro models, are already known from the literature data; evasion from apoptosis, alteration of nucleotide metabolism and drug transporters, the transition from an epithelial to a mesenchymal phenotype, and the activation of the stem compartment are some of the mechanisms directly related to the resistance and to a greater aggressiveness of cancer cells [22].

The phenomenon of resistance is generally studied, taking into account the pathology and the type of drug, hindering the identification of a unique panel of genes and pathways. To identify the characteristics of our model, we tested a commercial panel of 168 genes, 84 involved in DR and 84 in the EMT, identifying 99 deregulated genes. By focusing our attention on the highest up-regulated ones, we found the AHR/ARNT axis overexpressed in chemotherapy-resistant choriocarcinoma stem cells [23]. PPARG, up-regulated in our model, has already been shown to be associated with gemcitabine resistance in CCA; resistance is mediated by the overexpression of miR-130a-3p [24]. In 82.3 cells, we observed overexpression of HIF1A; in medulloblastoma, the upregulation of HIF1A correlated with a worse response to cyclophosphamide through the inhibition of a gene of the cytochrome p450 family, CYP3A5 [25], also found to be down-regulated in our model. The high expression of CAMK2N1 and MMP9, on the other hand, has already been described in xenopatients of ovarian cancer treated with cisplatin. Elevated TGFB1-2 levels are correlated with increased aggression in triple-negative breast cancers; their inhibition also decreases the expression of VIM and FN1, genes found up-regulated in our model [16].

These are just some of the possible genes involved in the resistance mechanism; further studies should be conducted to verify their current and real involvement and to evaluate their possible role as new targets for therapy. In addition, other events, such as methylation, the expression of miRNA, or long non-coding RNA that indirectly altered the response to drugs, may contribute to resistance.

From mRNA sequencing analysis, we found that up-regulated genes are enriched for vasculature development, ECM remodeling, cell adhesion, inflammation, and EMT processes, while down-regulated genes are associated with ion and membrane transport, bile acid and xenobiotic metabolism, tissue homeostasis, and cell surface receptor signaling pathways, underlined an aggressive phenotype of 82.3 cells.

Our model, as demonstrated by the karyotype, appears to consist of three different clones; a first step could be subcloning the cell line to verify the different sensitivity/resistance to drugs, the different proliferative and stem cell capacity of the clones. A heterogeneous population is actually the mirror of the tumor, which is not made up of a single cell clone. The pressure of the drug could, in fact, eliminate the most sensitive cells, but at the same time, it could favor the expansion of resistant clones.

## 4. Materials and Methods

### 4.1. Establishment of ICC Cell Line from a PDX

The surgical sample was obtained from an iCCA Italian patient, treatment-naïve, subjected to surgical resection. Informed consent was obtained from the patient involved in the study, following the protocol approved by the institutional ethics committee (PROFILING protocol: “Prospective study for the determination of molecular profiles of patients affected by tumors resistant to target therapies.”; Ethic code: 001-IRCC-00IIS-10, version 6.1, FPO-IRCCS, 23 June 2016; 001-IRCC-00IIS-10 version 6.1, FPO-IRCCS, Candiolo Cancer Institute, Turin, Italy). Furthermore, written informed consent has been obtained from the patient to publish this paper. For PDX establishment, a fragment of fresh tumor tissue (4 × 4 mm) was subcutaneously (s.c.) implanted in the flank of NOD/SCID (non-obese, diabetic, severe acquired immunodeficiency) mice. All animal procedures were approved by the Institutional Ethical Committee for Animal Experimentation (Fondazione Piemontese per la Ricerca sul Cancro) and by the Italian Ministry of Health (Ethic code: 177/2015-PR; 178/2015-PR and 106/2021-PR). Tumor mass growth was monitored weekly and measured by caliper. On reaching 1 cm^3^ of volume, the tumor was explanted from the first generation and implanted in other animals. The implants were carried out up to the fourth generation, which is conventionally considered the time of PDX stabilization. Tumor at the fourth generation was explanted and enzymatically dissociated with collagenase (200 U/mL) for 3 h at 37 °C, then inactivated by fetal bovine serum (FBS). Cells suspension was filtered and then cultured in DMEM/Knockout/F-12 culture medium with 10% FBS, penicillin, and streptomycin (P/S) at a cell density of 300,000/mL and incubated at 37 °C in an atmosphere with 95% air and 5% CO2 (all reagents were from Sigma-Aldrich, St. Louis, MO, USA). The medium was replaced twice a week. When the cells reached 70–80% of confluence, they were propagated in the optimal culture condition. After one year (at the sixtieth passage), a stable cell line, named 82.3, was obtained.

### 4.2. Cell Lines

HuH28 iCCA cell line (Cell Bank, RIKEN Bioresource Center Riken Cell Bank, Ibaraki, Japan) was cultured in RPMI 1640 containing 10% FBS (all from Sigma-Aldrich, St. Louis, MO, USA), 100 U/mL penicillin, and 100 μg/mL streptomycin (P/S; Life Technologies, Gaithersburg, MD, USA). MT-CHC01 cells (26486326) were cultured in DMEM/Knockout/F-12 culture medium with 10% FBS and P/S.

### 4.3. Flow Cytometry Analysis

Immunophenotyping of 82.3 cells was performed by flow cytometric analysis. Cells were washed in 1× PBS containing 0.1% bovine serum albumin (BSA, Sigma-Aldrich, Saint Louis, MO, USA) and 0.01% sodium azide. For intracellular markers, cells were permeabilized with fix and perm reagent (BD Italia) following the manufacturer’s instructions. The following antibodies were used: FITC (Fluorescein isothiocyanate) conjugated mouse mAbs anti-CK7 and anti-CK19 (Abcam, Cambridge, UK) and anti-CD44 (BD Bioscience, San Jose, CA, USA), APC-(allophycocyanin) conjugated mouse mAbs anti-EPCAM and anti-AFP (BD Bioscience Europe), anti-CD34 and anti-CD133 (Miltenyi Biotec S.r.l., Bologna, Italy), PE (phycoerytrin) conjugated mAbs anti-CD24, anti-CXCR4, anti-Oct3/4, anti-FOXA1/2, anti-PDX1, (all from BD), anti-CD338 (R&D Systems, Inc., Minneapolis, MN, USA), PerCP-Cy 5.5 conjugated mAbs anti-SOX2/17, Alexa Fluor 647 conjugated mAbs anti-Nanog, anti-Stro1, and Alexa Fluor 488 conjugated mAbs anti-PAX6 (BD Bioscience Europe).

### 4.4. Immunofluorescence Staining

For immunofluorescence, 82.3 cells at a density of 5 × 10^3^ were plated in 8-well chamber slides (BD Falcon, Glendale, AZ, USA). Briefly, after 24 h, cells were fixed with 4% paraformaldehyde for 20 min and permeabilized with ice-cold methanol for 10 min. After saturation with PBS-1% bovine serum albumin (BSA), cells were incubated overnight with anti-EPCAM Alexa Fluor 488 Conjugate (1:50 Cell Signaling, #5198), anti-CK19 FITC Conjugate (1:50 Abcam, #ab87014), and anti-CK7 Alexa Fluor 555 Conjugate (1:50 Abcam, #ab209601). Nuclei were counterstained with DAPI. Images were acquired by confocal microscopy (Nikon, Lipsi, Tokyo, Japan) at 40× magnification.

### 4.5. Population Doubling Time

The population doubling time was determined by seeding 1.5 × 10^5^ cells in the optimal medium in 24-well plates in triplicate. Viable cells were counted in a hemacytometer/Burker chamber by staining with trypan-blue at 24, 48, and 72 h after seeding. The average number of cells was calculated in three different experiments.

To calculate the population doubling time (DT), we used the following formula: DT = T ln2/ln(Xe/Xb), in which T is the time duration of culture, Xe is the cell number at the end of the incubation time, and Xb is the cell number at the beginning of the incubation time.

### 4.6. Drugs

Gemcitabine and oxaliplatin were obtained, respectively, from SANDOZ (Novartis Division, Siena, Italy) and SUN Ranbaxy (Sun Pharmaceutical Industries Ltd., Goregaon, Mumbai, India); 5-FU and carboplatin were obtained from TEVA (Teva Italia srl, Milano, Italy). All drugs were dissolved in water for injection and aliquoted at a different working solution to avoid degradation.

### 4.7. Cell Growth Assay

Cell growth assay was performed by seeding 2500 cells per well into 96-well plates in an optimal culture medium. After 24, 48, and 72 h, cell viability was evaluated with the Cell Titer-Glo^®^ commercial kit (Promega Italia, Milan, Italy) following the manufacturer’s protocol. The measurement of luminescence was performed through the Glomax microplate reader (Glomax-Multi Detection System, Promega, Milan, Italy). The antitumor activity of different chemotherapeutic agents (gemcitabine, 5-FU, carboplatin, and oxaliplatin) was also investigated, treating cells with escalating doses of drugs. The IC50 of the drug (concentration of drug needed to inhibit the 50% of growth) was calculated using Calcusyn software (Biosoft, Cambridge, U.K.), based on the Chou–Talalay method.

### 4.8. Apoptosis Assay

For the apoptosis analysis, 1 × 10^5^ cells of MT-CHC01 and 2 × 10^5^ cells of 82.3 were plated in 6-well plates. Cells were treated after 24 h with gemcitabine (30 µM), 5-FU (384 µM), oxaliplatin (20 µM), and carboplatin (40 µg/mL). Quantification of apoptotic cells was evaluated after 72 h by the Annexin V-APC (allophycocyanin)/propidium iodide (PI) staining assay. In particular, after drug treatment, the supernatant containing detached death cells and 2 × 10^5^ cells were recovered. The cells were then incubated for 30 min at 4 °C in the dark in 500 µL of commercial binding buffer (Bender MedSystems, Wien, Austria). After washing in PBS, the cells were resuspended in binding buffer containing Annexin V-APC (Bender MedSystem) and PI 50 µg/mL (ThermoFisher Scientific, Waltham, MA, USA) and then incubated for 1 h at 4 °C in the dark. The samples were then acquired by a flow cytometer. For each experiment, 30,000 events were acquired. To express cell death, the average of the percentages of death cells (early and late apoptotic) obtained in the two independent experiments was calculated.

### 4.9. Sphere-Formation Assay

The cholangiosphere formation assay was carried on seeding 1.5 × 10^5^ of 82.3-ICC cells per well into 6-well plates (ultra-low attachment surface) and cultured in stem cell medium serum-free conditions (DMEM-F12 medium, 1X B27, 20 ng/mL human EGF, 10 ng/mL human FGF, 0.4% BSA, 4 μg/mL insulin and P/S). Sphere formation was monitored on days 7, 10, and 14 after seeding.

### 4.10. In Vitro Migration Assay

Migration potential was investigated by transwell chambers assay (1 cm^2^/well, BD Falcon). The upper and lower cultures were separated by an 8-µm pore size poly-vinyl-pyrrolidone-free polycarbonate filters (BD Falcon). The experiments were carried out in triplicates.

After the incubation period, filters were fixed with methanol and stained with 0.5% crystal violet in 25% methanol; cells on the upper surface of the filters were removed using cotton swabs. Cells invading the lower surface were counted in five random fields and expressed as the number of invading cells per well. Student’s *t*-test was used to analyze migration data (CI: 0.95%, *p* < 0.05 as statistically significant).

### 4.11. Tumorigenicity in NOD/SCID Mice

For in vivo studies, NOD (non-obese diabetic)/Shi-SCID (severe combined immunodeficient) female mice (4–6 weeks old) (Charles River Laboratory) were maintained under sterile conditions in micro-isolator cages at the animal facilities of IRCCS-Candiolo. All animal procedures were approved by the Institutional Ethical Committee for Animal Experimentation (Fondazione Piemontese per la Ricerca sul Cancro) and by the Italian Ministry of Health (Ethic code: 177/2015-PR; 178/2015-PR and 106/2021-PR).

In three independent experiments, six mice were s.c. injected into the right flank under anesthesia (mixture of isoflurane and nitrous oxide) with 3.0 × 10^6^ 82.3 cells in 50% growth factor-reduced BD Matrigel (BD Biosciences, San Jose, CA, USA). Tumor diameters were measured weekly after cell injection up to 20 weeks.

### 4.12. DNA Identify Analysis by Fingerprinting

Genomic DNA was isolated from cells by using the Wizard^®^ Genomic DNA Purification Kit (Promega, Milan, Italy). DNA fingerprinting was performed by the analysis of short tandem repeats (STR) at 16 different loci (D5S818, D13S317, D7S820, D16S539, D21S11, vWA, TH01, TPOX, CSF1PO, D18S51, D3S1358, D8S1179, FGA, Penta D, Penta E, and amelogenin). Amplicons derived from multiplex PCRs were separated by capillary electrophoresis (3730 DNA Analyzer, Applied Biosystems, Foster City, CA, USA) and analyzed using GeneMapper v.5 software GeneMapper v.5 (ThermoFisher Scientific, Waltham, MA, USA).

### 4.13. Karyotype Analysis

The cell line was subjected to chromosome analysis by G-banding. Cells were trypsinized with trypsin/EDTA after treatment with colchicine (colcemid 10 µg/mL) for 1–3 h, and the slides were prepared according to standard methods. Briefly, cells were incubated in 0.075 M hypotonic KCl solution for 20 min and fixed in glacial acetic acid-methanol (3:1). G-banding was performed using 2 × SSC at 65 °C for 2 min and Wright’s stain for 2 min (all reagents were from Sigma-Aldrich, St. Louis, MO, USA). Metaphase images were acquired using an Olympus BX61 microscope (Olympus Corporation, Tokyo, Japan) and analyzed by CytoVision software 7.2 (Leica Biosystems, Newcastle Ltd., UK). A mid-range resolution of 300 bands was achieved. The anomalies were described according to the International System for Human Cytogenetic Nomenclature, 2016 [26].

### 4.14. RNA Extraction, RT2PCR Array for Epithelial to Mesenchymal Transition (EMT) and Drug Resistance and RNA Sequencing

Total RNA was extracted from cells at 70% of confluence. After washing in PBS, cells were subjected to phenolic extraction by lysis with the TriReagent and 200 µL of chloroform. After stirring for 15 s and incubating at room temperature for 5 min, the lysate was centrifuged for 20 min at maximum speed at 4 °C. The RNA recovered in the aqueous phase was purified by ion exchange resins using the Absolutely RNA miRNA kit (Agilent Technologies, Santa Clara, CA, USA). The residual DNA was eliminated by digestion with DNase I, directly on resin. The RNA was then washed and eluted in nuclease-free water. Total RNA quality and quantity were checked by using Bioanalyzer and Qubit, respectively. A total of 500 ng of tot-RNA were reverse transcribed using the RT2 First-strand kit (Qiagen). The qRT-PCR reaction was performed using the 2X SYBR Green Mastermix. Ninety-six-well plates (RT2 PCR array, Qiagen, Hilden, Germany) were used, each containing 84 genes specifically associated with EMT and drug resistance processes. The expression values were calculated with the ΔΔCt method; using, for their stability in terms of expression, ACTB and HPRT1 as housekeeping genes. Since the 82.3 cells lack a sensible counterpart, the MT-CHC01 cells were used as reference. The experiment was conducted in triplicate. Libraries preparation was performed using TruSeq RNA Library Preparation Kit v2 following the manufacturer’s instructions (Illumina, San Diego, CA, USA). Libraries were sequenced on the Illumina NextSeq 550 system. Quality control on raw sequence files (fastq) was performed with FastQC 0.11.9. Trimming was performed with Cutadapt 2.9. STAR 2.6.1d was used for the alignment step against the human GRCh38 reference genome, and reads were counted with FeatureCounts 1.6, asking for a gene-level report. Finally, the EdgeR package, available within Bioconductor, (https://www.bioconductor.org version 3.12), was used to perform a TMM normalization on count data and to determine differential expression. Raw data were deposited on GEO Omnibus (https://www.ncbi.nlm.nih.gov/geo/, GSE171148 available from 1 May 2021).

### 4.15. Statistical Analysis

A two-way ANOVA test was used to analyze cells and tumor growth and drug response; a *p*-value less than 0.05 (confidence interval 95%) was considered statistically significant. The one-way ANOVA was used to compare the response at different doses of chemotherapeutic agents within the same cell line considering as statistically significant a *p*-value less than 0.05 (confidence interval 95%). Data are mean and standard deviation (SD) (bars) of values from at least triplicate assays. Statistical analysis was performed with Student’s *t*-test. Asterisks indicate a significant *p*-value (* *p* < 0.01, ** *p* < 0.001, *** *p* < 0.0001, **** *p* < 0.00001).

## 5. Conclusions

In conclusion, we have stabilized a new human intrahepatic cholangiocarcinoma cell line of Caucasian origin that could better recapitulate the characteristics of this tumor in relation to the etiology and Western ethnicity. This model could provide a useful preclinical tool to study the mechanisms of cross-resistance and the identification of alternative therapies for this tumor type.

## Figures and Tables

**Table 1:** DNA fingerprinting of 82.3 cells.

Locus	82.3			
	Allele 1	Allele 2	>Allele 3	>Allele 4
AMEL	X	Y	-	-
CSF1PO	11	-	-	-
D13S317	9	10	-	-
D16S539	11	-	-	-
D18S51	13	16	-	-
D21S11	30	-	-	-
D3S1358	14	15	-	-
D5S818	12	-	-	-
D7S820	8	9	-	-
D8S1179	13	14	-	-
FGA	23	-	-	-
Penta D	9	-	-	-
Penta E	10	16	-	-
TH01	9	-	-	-
TPOX	9	-	-	-
vWA	18	-	-	-

**Table 2:** Genes related to the “drug resistance” process modulated in the multidrug-resistant 82.3 cells compared to the sensitive MT-CHC01.

Up-Regulated Genes	Average Fold Change	SD	Down-Regulated Genes	Average Fold Change	SD
*AHR*	152.60	4.39	*ABCC1*	0.11	0.04
*APC*	8.80	1.82	*ABCC2*	0.04	0.01
*ARNT*	3.06	0.46	*ABCC3*	0.33	0.08
*BCL2*	2.22	0.49	*ABCC5*	0.25	0.08
*BRCA2*	11.43	1.41	*BAX*	0.46	0.07
*CCND1*	1.67	0.44	*BCL2L1*	0.09	0.03
*CDKN1A*	29.58	0.44	*CCNE1*	0.26	0.03
*CYP2E1*	6.37	1.99	*CDK2*	0.41	0.07
*EGFR*	5.15	1.60	*CDK4*	0.28	0.14
*EPHX1*	1.69	0.51	*CDKN1B*	0.27	0.15
*HIF1A*	7.07	0.87	*CLPTM1L*	0.07	0.02
*MET*	3.16	0.40	*CYP3A5*	0.11	0.03
*MSH2*	5.66	1.25	*ELK1*	0.01	0.01
*MYC*	1.74	0.19	*ERBB2*	0.16	0.08
*NAT2*	10.82	0.17	*GSK3A*	0.20	0.06
*PPARA*	2.19	0.31	*GSTP1*	0.40	0.13
*PPARG*	9.29	1.41	*MVP*	0.15	0.07
*RB1*	5.11	0.74	*NFKB2*	0.22	0.00
*SOD1*	1.69	0.17	*NFKBIB*	0.27	0.07
*TOP2A*	3.91	0.54	*RARA*	0.39	0.07
*TOP2B*	16.13	1.66	*RARG*	0.18	0.05
*TPMT*	6.25	0.01	*RELB*	0.36	0.08
*UGCG*	6.69	0.43	*RXRA*	0.18	0.04
*-*	-	-	*RXRB*	0.49	0.14
-	-	-	*SULT1E1*	0.34	0.04
-	-	-	*TP53*	0.07	0.02

**Table 3:** Genes related to the “epithelial to mesenchymal transition” process modulated in the multidrug-resistant 82.3 cell line compared to the sensitive MT-CHC01.

Up-Regulated Genes	Average Fold Change	SD	Down-Regulated Genes	Average Fold Change	SD
*CALD1*	3.51	0.12	*AKT1*	0.17	0.03
*CAMK2N1*	14.79	3.50	*BMP2*	0.05	0.02
*CAV2*	2.17	0.84	*ESR1*	0.33	0.09
*CDH2*	5.47	1.08	*F11R*	0.19	0.04
*COL5A2*	3.62	1.07	*FOXC2*	0.05	0.00
*DSC2*	11.94	2.90	*IGFBP4*	0.02	0.01
*DSP*	6.91	0.55	*ILK*	0.23	0.01
*EGFR*	5.92	0.59	*ITGA5*	0.01	0.00
*FGFBP1*	6.46	2.95	*KRT19*	0.28	0.01
*FN1*	31.61	6.18	*KRT7*	0.37	0.13
*FZD7*	2.10	0.41	*MAP1B*	0.13	0.01
*GSK3B*	5.43	0.34	*MSN*	0.15	0.04
*IL1RN*	4.31	0.50	*MST1R*	0.07	0.01
*ITGAV*	3.73	0.16	*NOTCH1*	0.10	0.03
*ITGB1*	4.55	0.29	*PPPDE2*	0.36	0.00
*MMP9*	29.40	0.13	*SPP1*	0.16	0.01
*OCLN*	5.93	0.06	-	-	-
*PLEK2*	2.19	0.29	-	-	-
*PTK2*	2.02	0.51	-	-	-
*PTP4A1*	2.89	0.03	-	-	-
*RAC1*	13.17	1.75	-	-	-
*SIP1*	2.58	0.35	-	-	-
*SMAD2*	4.04	0.45	-	-	-
*STEAP1*	2.78	0.47	-	-	-
*TCF4*	6.10	0.26	-	-	-
*TGFB1*	2.09	0.61	-	-	-
*TGFB2*	89.35	16.11	-	-	-
*TGFB3*	1.82	0.30	-	-	-
*TIMP1*	1.67	0.14	-	-	-
*TMEFF1*	12.51	4.12	-	-	-
*TSPAN13*	5.72	0.59	-	-	-
*VCAN*	168.97	28.95	-	-	-
*VIM*	504.06	83.12	-	-	-
*VPS13A*	9.95	0.97	-	-	-

## Data Availability

The data presented in this study are openly available in GEO Omnibus (https://www.ncbi.nlm.nih.gov/geo/, GSE171148 available from 1 May 2021).

## Supplementary Materials

The following are available online at https://www.mdpi.com/article/10.3390/cancers13092051/s1, Figure S1: Immunophenotyping of 82.3 cells at early passage, Figure S2: AFP expression in 82.3 cells by Flow Cytometry, Figure S3: Growth curves of 82.3 cells at early passage, Table S1: Differentially expressed genes in 82.3 cells vs. MT-CHC01 cells, Table S2: (A) Pathway maps enriched for up-regulated genes. (B) Process networks enriched for up-regulated genes, Table S3: (A) Pathway maps enriched for down-regulated genes. (B) Process networks enriched for down-regulated genes.
