# Supplementary material for: A Novel Multidrug-Resistant Cell Line from an Italian Intrahepatic Cholangiocarcinoma Patient
Source: Cancers (Basel). 2021 Apr 23;13(9):2051. doi: 10.3390/cancers13092051 (PMC8123022; doi:10.3390/cancers13092051)
Supplement: Supplementary file 1 [file cancers-13-02051-s001.zip › cancers-1197383-supplementary/cancers-1197383-supplementary.pdf]

Supplementary Materials: A Novel Multidrug-Resistant Cell Line from an Italian Intrahepatic Cholangiocarcinoma Patient

Caterina Peraldo-Neia, Annamaria Massa, Francesca Vita, Marco Basiricò, Chiara Raggi, Paola Bernabei, Paola Ostano, Laura Casorzo, Mara Panero, Francesco Leone, Giuliana Cavalloni and Massimo Aglietta

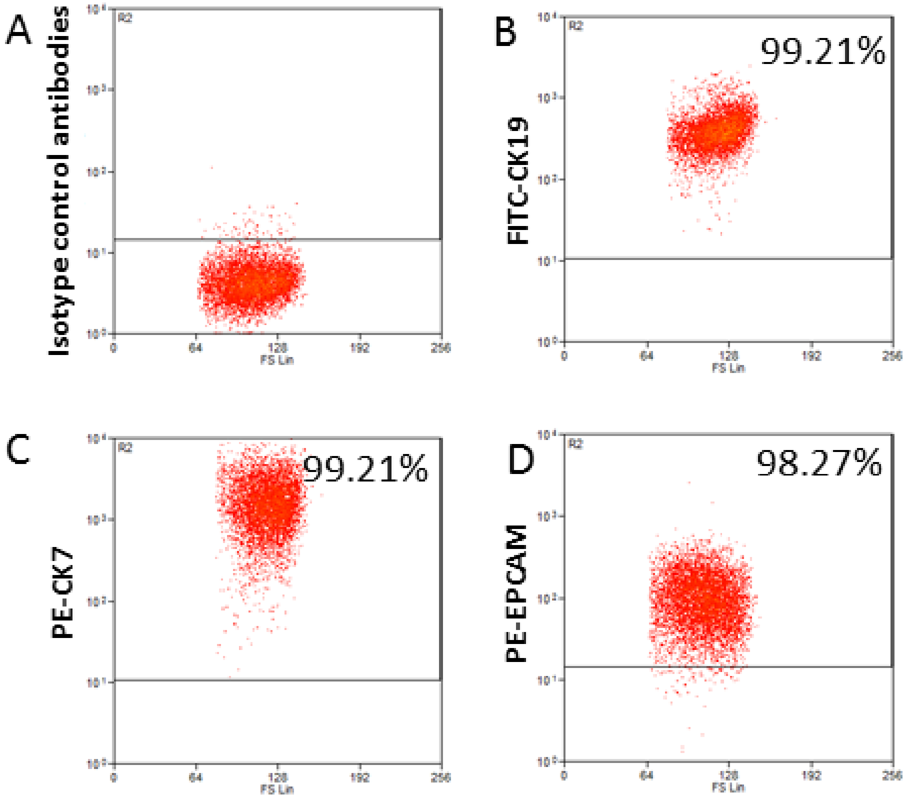

Figure S1. Immunophenotyping of 82.3 cells at early passage. Negative control: cells incubated with antibody isotype as primary antibody (**A**). Epithelial cell markers CK19 (99.21%) (**B**), CK7 (99.21%) (**C**) and EPCAM (98.27%) (**D**).

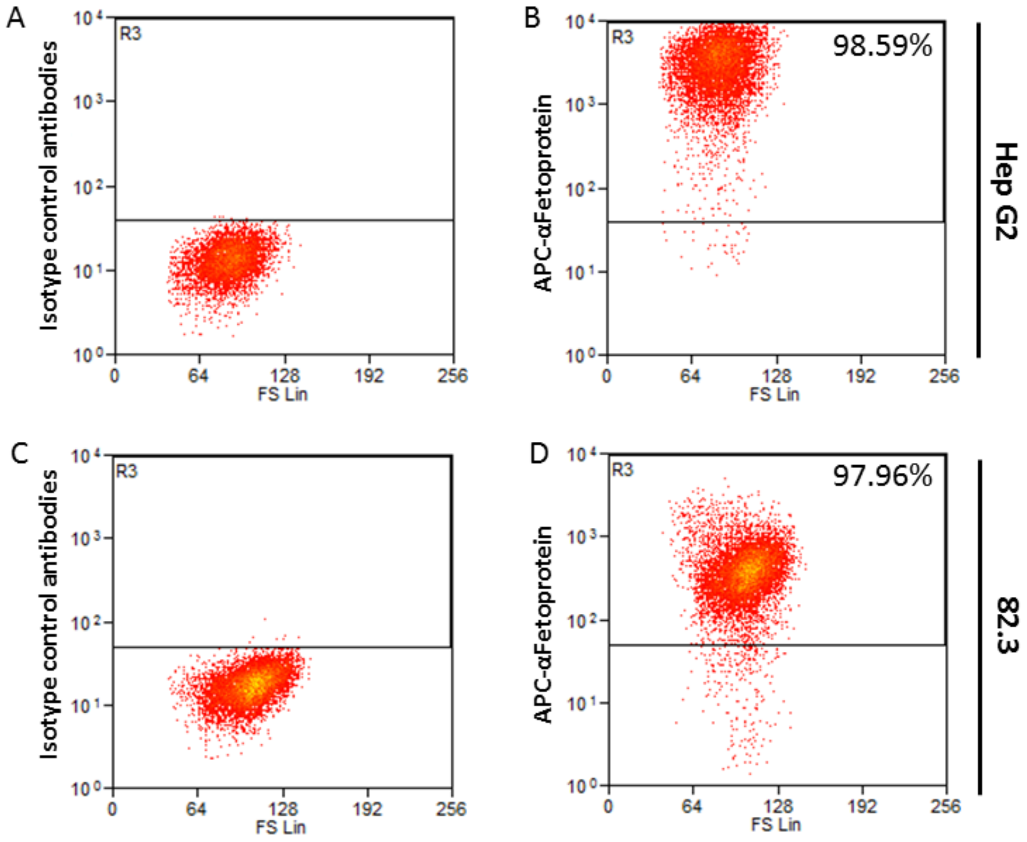

Figure S2. AFP expression in 82.3 cells by Flow Cytometry. Negative control: cells incubated with antibody isotype as primary antibody (**A**, **C**). Alpha-fetoprotein expression in 82.3 (97.96%) (**D**) and Hep G2 positive control cell line (98.59%) (**B**).

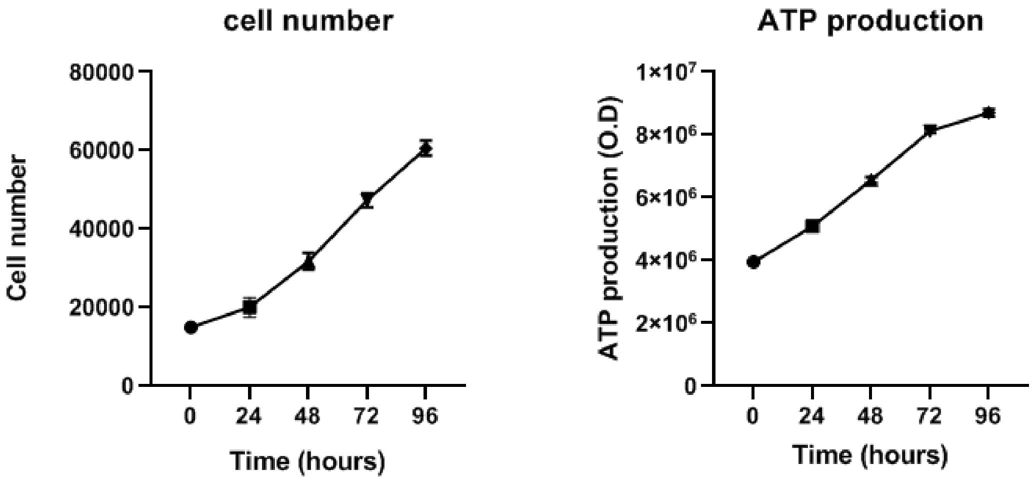

Figure S3. Growth curves of 82.3 cells at early passage. Viable cells were counted at 24, 48, 72 and 96 hours after seeding (**A**). ATP production at 24, 48, 72 and 96 hours of culture after seeding, obtained by CellTiter GLO® assay of 82.3 cells (**B**).

Table S1. Differentally expressed genes in 82.3 cells vs MT-CHC01 cells.

Table S2. (**A**) Pathway maps enriched for up-regulated genes. (**B**) Process networks enriched for up-regulated genes.

Table S3. (**A**) Pathway maps enriched for down-regulated genes. (**B**) Process networks enriched for down-regulated genes.

Table S1–S3. are provided separately, attached as Excel Files.
